# Acute Pancreatitis: A Rare Cause of Complement-Mediated Thrombotic Microangiopathy

**DOI:** 10.7759/cureus.36896

**Published:** 2023-03-30

**Authors:** Jonathan Livingston, Gurneel Dhanesar

**Affiliations:** 1 Internal Medicine, New York Medical College, Denville, USA

**Keywords:** general internal medicine, complement mediated hemolytic anemia, atypical hus, cm-tma, pancreatitis

## Abstract

Disruption of the complement regulatory system can provoke thrombotic microangiopathy (TMA), leading to clinical manifestations of generalized fatigue from hemolytic anemia, purpura caused by thrombocytopenia, and acute kidney injury from end-organ ischemia. This particular classification of TMA is known as complement-mediated thrombotic microangiopathy (CM-TMA). In CM-TMA, an inciting event such as infection, surgery, vaccination, or pregnancy triggers an inflammatory response resulting in the expression of inherited mutations or the development of autoantibodies against complement regulatory proteins, which leads to microangiopathic hemolytic anemia, thrombocytopenia, and direct damage to renal endothelial cells. The diverse etiologies of CM-TMA make diagnostic and therapeutic decisions a challenging endeavor. We encountered a young male patient who presented with significant lethargy and confusion. The initial diagnosis was consistent with systemic inflammatory response syndrome secondary to acute pancreatitis; however, the hospital course was complicated by subsequent acute renal failure, hemolytic anemia, and thrombocytopenia, likely indicating CM-TMA. The patient was successfully treated with plasma exchange therapy and eculizumab. We suspect that our patient likely developed CM-TMA from an episode of acute pancreatitis. Prompt diagnosis and early intervention are essential to improving morbidity and mortality. This is underscored by the development of monoclonal antibody therapy that directly targets the pathogenic complement proteins, which have been shown to improve renal disease outcomes.

## Introduction

Complement-mediated thrombotic microangiopathy (CM-TMA) is a form of microangiopathic hemolytic anemia and thrombocytopenia with acute kidney injury characterized by a complex syndrome of diverse etiologies that make diagnostic and therapeutic decisions a challenging endeavor. Formerly referred to as 'atypical hemolytic uremic syndrome', CM-TMA has been reclassified to more accurately depict the etiology of the underlying disease process. The current incidence of CM-TMA was thought to be significantly underreported prior to the discovery of its distinct disease process; however, it has an estimated prevalence of seven per one million children. These data are further confounded by recent literature describing that 50% of cases of CM-TMA are diagnosed in adults. Most cases of CM-TMA are due to genetic mutations in the proteins responsible for regulating the alternative complement pathway, with as few as 8-10% of cases being caused by autoantibodies directed at complement proteins. The phenotypic expression of these genetic variants or the activation of autoantibodies is presumed to be triggered by an inciting event such as infection, surgery, or pregnancy. We present a case of CM-TMA likely precipitated by acute pancreatitis.

## Case presentation

**Figure 1:**
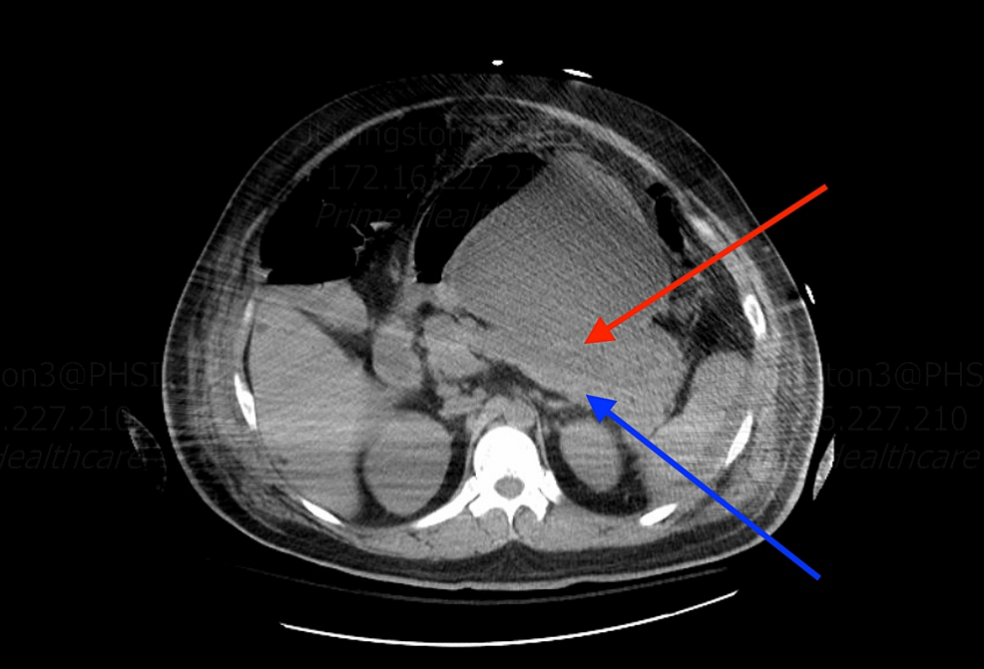
Non-contrast CT scan of the abdomen and pelvis The body of the pancreas appears atrophic (blue arrow). There is a large loculated fluid collection seen around the body and tail of the pancreas abutting the greater curvature of the stomach consistent with pancreatic pseudocysts (red arrow).

A 38-year-old male with no reported past medical history was brought to the hospital via emergency medical services after being found by his mother to be lethargic and confused. Collateral information obtained from the mother included no history of alcohol use or polysubstance abuse, recent travel, or viral infections. On physical examination, the patient was afebrile and somnolent, responsive only to painful stimuli, and the abdomen was moderately distended. Initial laboratory workup showed a non-anion gap acidosis, hyperglycemia with glucose measuring greater than 1,500 mg/dL (reference range: 70-140 mg/dL), a serum lipase level measuring 1,766 U/L (reference range: 13-60 U/L), and a C-reactive protein level measuring 42 mg/dL (reference range: 0.0-0.8 mg/dL). An abdominal ultrasound showed no biliary pathology or ductal dilation. An admitting diagnosis of systemic inflammatory response syndrome suspected secondary to acute idiopathic pancreatitis and a hyperosmolar hyperglycemic state was made. Shortly after the presentation, the patient became unresponsive, hypotensive with a systolic blood pressure in the 40s mmHg, and tachycardic with a heart rate of 140 beats per minute. The patient was intubated for airway protection, requiring mechanical ventilation and vasopressor support. The patient underwent a CT scan of the abdomen and pelvis to further characterize pancreatitis, which revealed pancreatic pseudocysts surrounding the body and tail of the pancreas (Figure 1).

This was managed initially with insulin infusion, bowel rest, and empiric meropenem in the setting of pseudocysts, and ultimately required a cystogastrostomy for drainage of the intra-abdominal fluid that did not grow bacteria. During the hospitalization, the patient developed acute renal insufficiency and became anuric, which necessitated renal replacement therapy. Concomitantly, the patient became anemic and thrombocytopenic, with a hemoglobin of 9.0 g/dL and a platelet count as low as 9 × 103/µL. Pertinent hematologic laboratory course results are summarized in (Table 1).

**Table 1:** Pertinent laboratory work up during hospital course Leukocyte reference range: 3.5-10.5 × 10^3^ µL. Hemoglobin reference range: 13.5-17.5 g/dL. Platelet count reference range: 150-450 × 10^3 ^µL. BUN reference range: 6-24 mg/dL. Creatinine reference range: 0.6-1.2 mg/dL. eGFR reference range: >60.

	Admission	Hospital day 4	Post plasma PEX
Leukocyte count (× 10^3^ µL)	23	14	16
Neutrophil count (%)	95%	80%	63%
Hemoglobin (g/dL)	17	9.6	8
Platelet count (× 10^3^ µL)	265	9	326
BUN (mg/dL)	62	51	42
Creatinine (mg/dL)	1.9	8.1	4
eGFR	34	10	17

Diagnostic workup was consistent with CM-TMA and included schistocytes on a blood smear, thrombocytopenia, acute renal insufficiency, and an ADAMTS13 level of 43%. Additionally, there was normal fibrinogen, lactate dehydrogenase of 1,831 U/l, haptoglobin of 336 mg/dL, negative HIV and hepatitis panels, a negative rheumatologic panel, an ADAMTS13 inhibitor factor less than 0.4 BU, and a Shiga-like toxin that was negative. The patient was treated with 12 days of plasma exchange and had a correction of platelet counts to within normal limits. The patient was eventually transferred to a tertiary-care facility for eculizumab therapy.

## Discussion

Complement-mediated thrombotic microangiopathy is a form of the microvascular disease known to cause microangiopathic hemolytic anemia and thrombocytopenia in addition to acute renal injury. It is distinct from the hemolytic uremic syndrome in that acute renal disease is not associated with diarrheal illness or Shiga toxin-producing bacteria [1]. CM-TMA is also delineated from the primary thrombotic microangiopathy, thrombotic thrombocytopenic purpura, by having normal levels of ADAMTS13 [2]. In contrast, CM-TMA is hypothesized to develop secondary to an inherited mutation in the genes responsible for regulating the complement pathway or the formation of autoantibodies against complement proteins [1,3]. These pathogenic mutations are thought to be expressed following an inciting event such as infection, surgery, or pregnancy that subsequently triggers an inflammatory response and activation of the complement cascade that occurs in patients with genetic predisposition or antibodies against complement proteins [3,4]. We suspect that our patient likely developed complement-mediated thrombotic microangiopathy from acute pancreatitis, which is a well-established yet uncommonly reported cause of TMA. In pancreatitis-induced TMA specifically, it is hypothesized that the release of inflammatory cytokines may play an additional role in the pathogenesis of CM-TMA by causing vascular endothelial damage and interfering with ADAMTS13 and Von Willebrand interactions, leading to platelet clumping and microthrombi formation [5]. The mainstay of treatment is plasma exchange therapy. This is followed by an immunosuppressive regimen for patients with complement antibodies that includes medications such as prednisone, cyclophosphamide, rituximab, and mycophenolate. In addition to renal transplantation, which appears to have significant recurrent disease, complement-modifying agents have now emerged. These include the anti-complement monoclonal antibodies eculizumab and ravulizumab which have been shown to improve renal outcomes in CM-TMA, further highlighting the importance of accurate diagnosis and distinction from alternative TMAs [6-7]. These antibodies inhibit the formation of the membrane attack complex that is assumed to mediate the microangiopathic changes and kidney injury in CM-TMA [1,6,8].

## Conclusions

Complement-mediated thrombotic microangiopathy is a form of microangioapthic hemolytic anemia and thrombocytopenia with acute renal injury characterized by a complex syndrome of diverse etiologies that make diagnostic and therapeutic decisions a challenging endeavor. Despite these challenges, precise evaluation and diagnosis are necessary to provide optimal treatment. Recently, there have been anti-complement agents approved specifically for the management of CM-TMA. The list of instigating factors continues to grow, and this case supports the possibility of acute pancreatitis precipitating complement-mediated thrombotic microangiopathy.
